# Prosthetic Rehabilitation of a Patient with Gastroesophageal Reflux Disease: 4-Year Followup

**DOI:** 10.1155/2014/270365

**Published:** 2014-03-09

**Authors:** Ricardo Coelho Okida, Daniela Micheline dos Santos, Aljomar José Vechiato Filho, Agda Marobo Andreotti, Rodrigo Antonio de Medeiros, Marcelo Coelho Goiato

**Affiliations:** ^1^Department of Restorative Dentistry, Aracatuba Dental School, Sao Paulo State University-UNESP, José Bonifácio, 1193 Vila Mendonça, 16015-050 Araçatuba, SP, Brazil; ^2^Department of Dental Materials and Prosthodontics, Aracatuba Dental School, Sao Paulo State University-UNESP, José Bonifácio, 1193 Vila Mendonça, 16015-050 Araçatuba, SP, Brazil

## Abstract

The gastroesophageal reflux disease (GERD) is one of the main causes of dental erosion. The aim of this case presented is to describe the prosthetic rehabilitation of a patient with GERD after 4 years of followup. A 33-year-old male patient complained about tooth sensitivity. The lingual surface of the maxillary anterior teeth and the cusps of the upper and lower posterior teeth presented wear. It was suspected that the feeling of heartburn reported by the patient associated with the intake of sports supplements (isotonics) was causing gastroesophageal changes. The patient was referred to a gastroenterologist and was diagnosed with GERD. Dental treatment was performed with metal-free crowns and porcelain veneers after medical treatment of the disease. With the change in eating habits, the treatment of GERD and lithium disilicate ceramics provided excellent cosmetic results after 4 years and the patient reported satisfaction with the treatment.

## 1. Introduction

Nowadays, the incidence of dental erosion has become a clinical reality [1, 2] with high prevalence in adult patients [3]. This alteration is defined as the constant loss of the teeth hard tissues caused by chemical agents without the influence of a carious process [2, 4]. The wear may cause the flattening of the occlusal surface and loss of occlusion vertical dimension of the patient. Additionally, there may be shortening of anterior teeth, bringing serious consequences to quality of life of the patient and preventing him from talking or smiling [5].

Among the causes of dental erosion, the gastroesophageal reflux disease (GERD) can be highlighted [4], which basically consists of an involuntary relaxation of the esophageal sphincter, allowing the return of the stomach acid to the oral cavity [6]. It is very important that the dentists recognize the signs of tooth erosion because this oral manifestation is one of the signs for diagnosing GERD [4, 7].

The modalities of rehabilitator treatment vary according to the degree of tooth wear [8, 9]. It is important to remove or treat the possible risk factors such that the properties of the restorative material are maintained [9–12] and rehabilitation does not become more complex [2].

The aim of this case presented is to describe the clinical manifestations of GERD, its diagnosis, and the medical and dental treatment of a patient with GERD after 4 years of followup.

## 2. Case Report

A 33-year-old male patient was admitted to Aracatuba Dental School-UNESP, complaining about tooth sensitivity to temperature variations caused by the ingestion of different foods and acidic substances.

**Figure 1:**
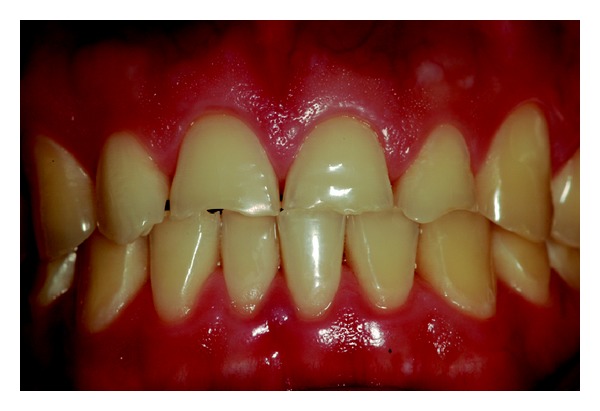
Facial view of dental erosion and shortening of the anterior teeth.

**Figure 2:**
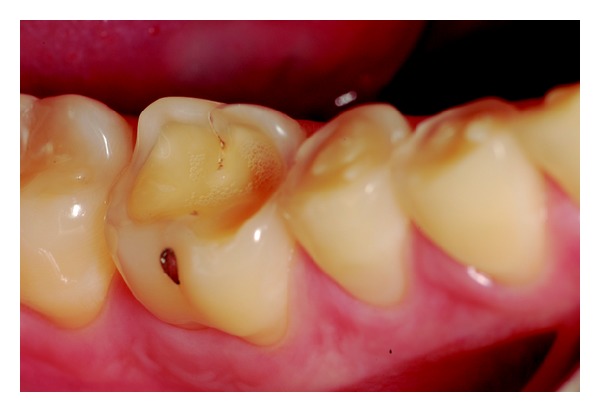
The posterior quadrant view of mandibular teeth with flatted occlusal surfaces.

**Figure 3:**
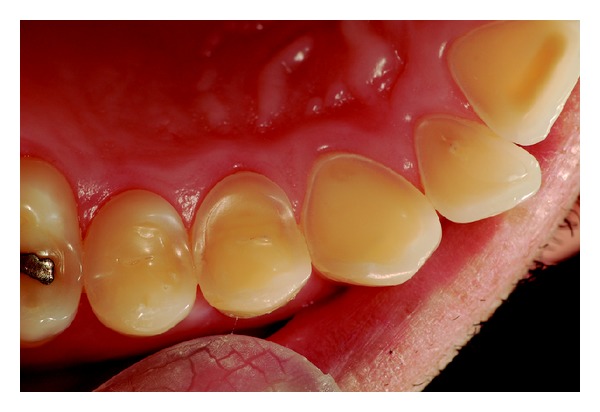
Occlusal view of maxillary teeth with worn surfaces.

**Figure 4:**
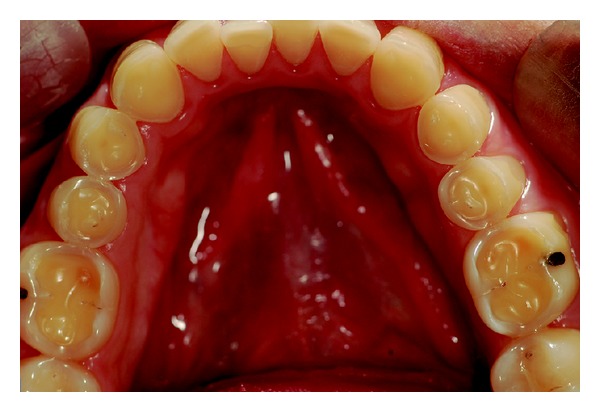
Mandibular arch view.

**Figure 5:**
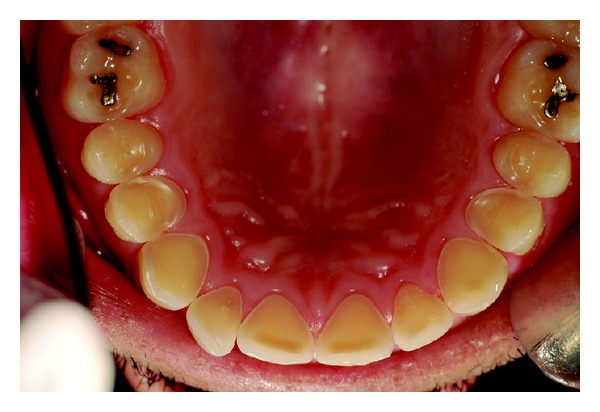
Maxillary arch view.

Wear on the lingual surface of the maxillary anterior teeth and the shortening of the cusps of upper and lower posterior teeth were observed. This change was just not observed in the lower anterior teeth. Slight reduction of occlusal vertical dimension of the patient was observed without the need of its restoration (Figures 1, 2, 3, 4, and 5). Thus, the anamnesis was performed to check the presence of parafunctional habits—which was discarded—and to identify the possible cause of tooth wear. The patient was unaware of the presence of systemic disorders and reported a feeling of heartburn. This symptom was probably caused by an exaggerated intake of sports supplements (isotonics) which might have caused the gastroesophageal changes since the patient practiced physical activity regularly.

The patient was referred to a gastroenterologist and the presence of hiatal hernia and gastroesophageal reflux disease was diagnosed after performing some specific tests. The medical treatment was based on the use of 40 mg of Omeprazol, twice a day for 30 days, and also change in eating habits such as the reduction of isotonic drinks and other foods that could exacerbate the symptoms. There was a decreased sensation of heartburn after medication treatment and only a regular medical followup was required.

The dental treatment could be performed once the symptoms were controlled and the patient was treating the disease with medicines. Initial photographs were taken and impressions of both arches were performed with stock trays and alginate (Hydrogum, Zhermack SpA Rovigo, Italy) to obtain the study models. The models were positioned on semiadjustable articulator for drafting and proposing the treatment plan.

**Figure 6:**
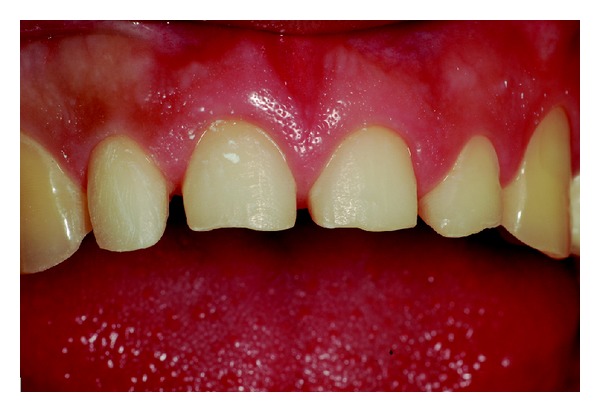
Frontal view of the maxillary prepared teeth.

**Figure 7:**
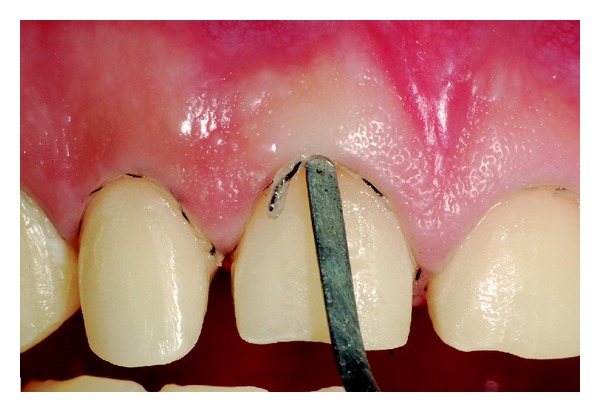
Insertion of gingival retractor cord.

**Figure 8:**
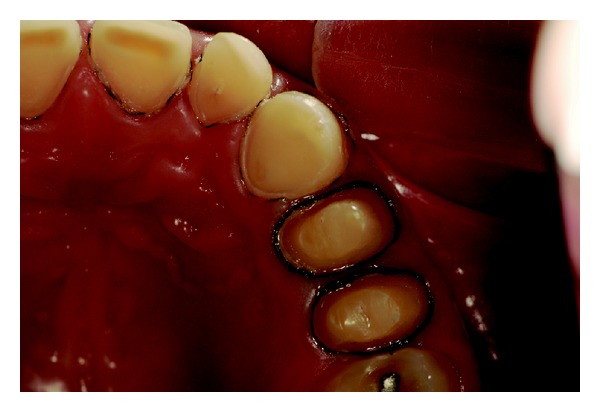
Cervical margins of the preparations exposed with gingival retractor cord.

Among the proposed treatment options, the patient chose to restore the dental anatomy with metal-free crowns and porcelain veneers. Initially, the affected teeth were prepared (Figure 6). Subsequently, impressions of the prepared teeth were made using silicone condensation in putty consistency and light body (Speedex, Vigodent SA Industria e Comercio, Bonsucesso, Rio de Janeiro, Brazil) and cervical margins of the preparations were exposed with gingival retractor cord number 000 (Ultrapack, Ultradent Products, Inc., South Jordan, Utah, USA) (Figures 7 and 8). Then, provisional restorations fabricated with indirect composite resin (Resilab, Wilcos do Brazil Industria e Comercio Ltda, Petropolis, Rio de Janeiro, Brazil) were cemented.

**Figure 9:**
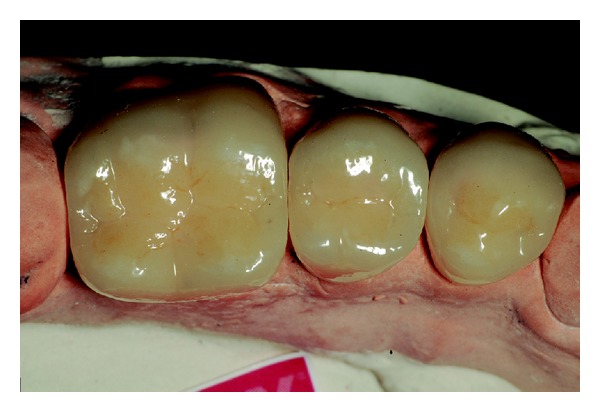
Laboratorial aspect of the posterior all-ceramic crowns.

**Figure 10:**
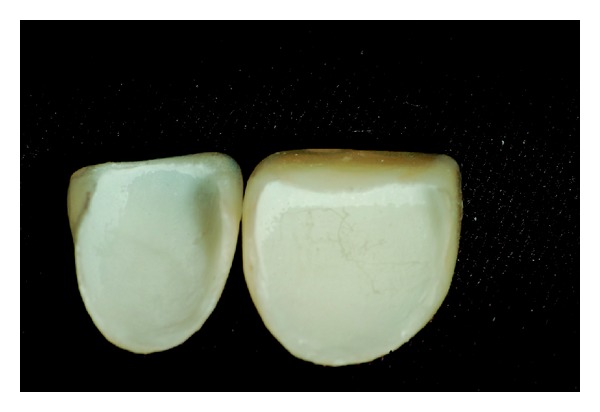
Clinical aspect of the conditioned inner face of the laminate veneers.

**Figure 11:**
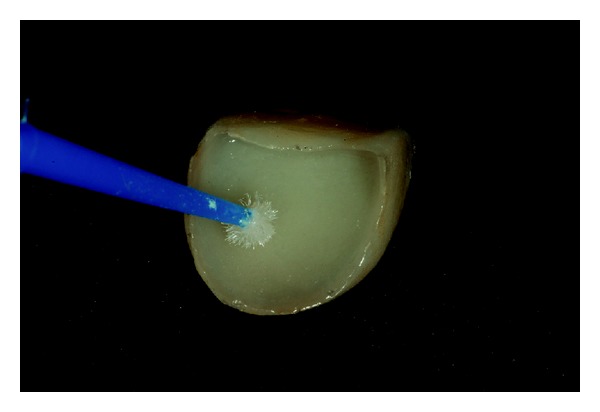
Clinical aspect of the conditioned inner face of the laminate veneers with silane layer applied.

The zirconia (IPS e.max ZirPress, Ivoclar Vivadent AG, Schaan, Liechtenstein) cores were confectioned and lithium disilicate ceramic was used as veneering ceramic for posterior crowns and laminate veneers (IPS e.max Ceram, Ivoclar Vivadent AG, Schaan, Liechtenstein) (Figure 9). The inner face of the laminate veneers was etched with 10% hydrofluoric acid (FGM Produtos Odontológicos, Joinville, Rio Grande do Sul, Brazil) for 20 seconds, rinsed with water, and dried with air and then a layer of silane was applied (Prosil, FGM Produtos Odontológicos, Joinville, Rio Grande do Sul, Brazil). The teeth were etched with 37% phosphoric acid (Dentsply, Petropolis, Rio de Janeiro, Brazil) for 30 seconds, rinsed with water, and dried and then a layer of adhesive was applied (Prime & Bond 2.1, Dentsply, Petrópolis, Rio de Janeiro, Brazil) (Figures 10 and 11).

**Figure 12:**
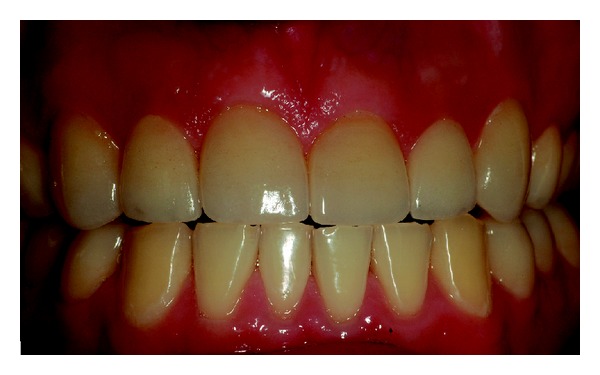
Final aspect after laminate veneers and all-ceramic crowns cementation.

The porcelain veneers were cemented only with base paste of resin cement (Variolink Veneer, Ivoclar Vivadent AG, Schaan, Liechtenstein), whereas the crowns were cemented with both pastes (base and catalyst) of dual resin cement (Variolink II, Ivoclar Vivadent AG, Schaan, Liechtenstein) (Figure 12).

**Figure 13:**
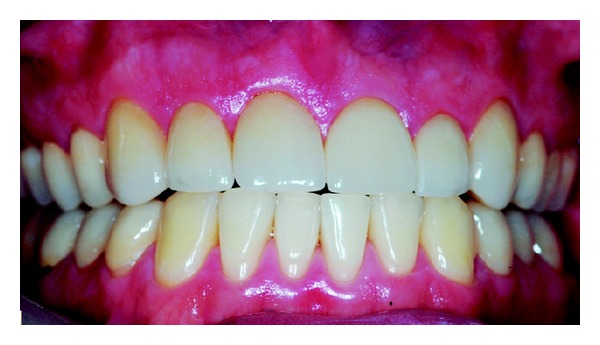
Clinical aspect of the restorations after 4 years of followup.

**Figure 14:**
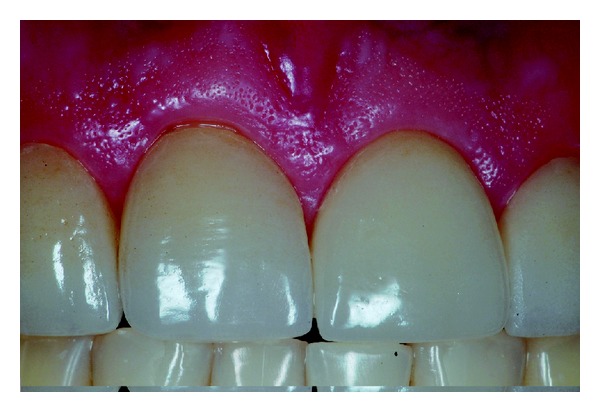
Healthy periodontal aspect after 4 years of restorations placement.

**Figure 15:**
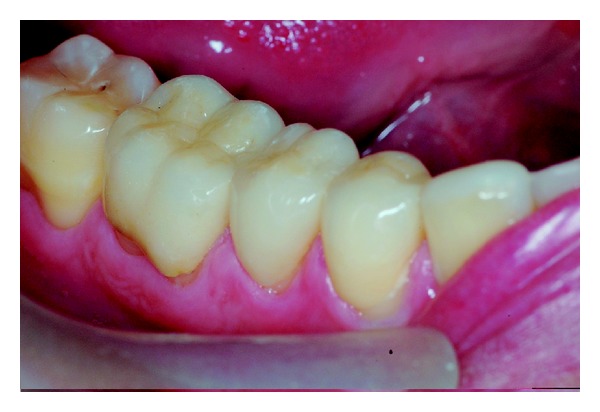
The posterior quadrant view of mandibular teeth with periodontal tissues free of gingival inflammation and no visible deteriorations of the restorations.

The patient reported no sensitivity after dental treatment and a homemade topical application of sodium fluoride gel 2% was recommended (Nupro Gel, Denstply, Petropolis, Rio de Janeiro, Brazil) every 15 days. After 4 years of followup the restorations showed no visible deterioration and the periodontal tissue was free of gingival inflammation (Figures 13, 14, and 15). The patient was satisfied with the treatment.

## 3. Discussion

Dental erosion has become an increasingly frequent and important clinical reality with multiple causes [8]. Among the risk factors for developing this problem are gastric pathologies and diets based on acidic foods. In this case presented, the tooth wear is caused mainly by GERD which produces an unintentional relaxation of esophageal sphincter, allowing the return of the stomach acid to the oral cavity [6]. The risk factors for GERD include obesity, hiatal hernia, and pregnancy [13].

We could confirm that the patient regularly practiced physical activities and made abusive use of acidic drinks as a supplement and their suspension was recommended by the gastroenterologist. People with healthy lifestyles may consume acidic drinks frequently in low salivation conditions (physical training) or making excessive use of the same day by day, trying to keep body weight [2].

Treatment for patients with GERD is to change eating habits and drug therapy to increase esophageal muscle activity or reduce the amount of stomach acid. In these cases, patients should avoid consumption of foods that irritate the gastric mucosa such as spicy and fatty foods, citrus fruits, coffee, tea, chocolate, alcohol, and soft drinks and get used to walk after meals [6].

Some studies show that ceramics are degraded on their surface when exposed to acidic solutions, which could compromise the longevity of these materials [10–12]. Thus, in this case, to reach a safe rehabilitation treatment, the pathology was treated and the patient's eating habits have been changed [9].

In order to achieve excellent cosmetic results associated with a good mechanical behavior, zirconia copings were chosen [14]. Likewise, lithium disilicate was used as veneering ceramic. The presence of 70% lithium disilicate crystals in its crystalline phase allows the reflection of light similarly to the natural tooth and provides a good flexural strength (360 to 400 MPa) to the restorative material [15].

## 4. Conclusion

Monitoring of patients and a multidisciplinary approach should be taken in rehabilitation treatments, since systemic changes might directly influence the final results of treatment. The modification of eating habits and treatment of GERD associated with the use of lithium disilicate ceramic offered excellent aesthetic result after 4 years and the patient reported satisfaction with the treatment.
